# Alectinib and Brigatinib: New Second-Generation ALK Inhibitors for the Treatment of Non–Small Cell Lung Cancer

**Published:** 2018-01-01

**Authors:** Tyler Beardslee, Justin Lawson

**Affiliations:** Emory Winship Cancer Institute, Atlanta, Georgia; and Emory University Hospital, Atlanta, Georgia

## Abstract

The treatment of non–small cell lung cancer (NSCLC) has been revolutionized by the discovery of genetic driver mutations and associated targeted therapies. Anaplastic lymphoma kinase (*ALK*) mutations are present in about 5% of NSCLC cases, and treatment with the first-generation ALK inhibitor crizotinib has shown better progression-free survival (PFS) and response rate compared to traditional chemotherapy. However, eventually, *ALK*-mutated NSCLC develops resistance to treatment with crizotinib, and second-generation ALK inhibitors such as ceritinib, brigatinib, and alectinib have been shown to be effective in the second-line setting after progression on crizotinib. In the second-line setting, alectinib showed an objective response rate (ORR) of 45% and PFS of 8 to 12 months. Brigatinib showed an ORR of 45% to 54% with a PFS of 9.2 to 12.9 months in the second-line setting. A more recent trial compared alectinib to crizotinib in the treatment-naive setting and showed a significant PFS benefit to treatment with alectinib. The second-generation ALK inihibitors brigatinib and alectinib offer new options for the treatment of *ALK* mutation–positive NSCLC.

Over the past several years, treatment for advanced and metastatic non–small cell lung cancer (NSCLC) has changed tremendously. Historically, NSCLC was treated primarily with chemotherapy, but advances over the past decade have allowed for treatment driven by molecular expression and genetic mutations of the tumor. The identification of anaplastic lymphoma kinase (ALK), epidermal growth factor receptor (EGFR), and ROS1 genetic aberrations has led to the development of treatments targeted at these oncogenic drivers. Rearrangements of ALK, a tyrosine kinase, have been found in about 5% of NSCLC cases (Soda et al., 2007; Takeuchi et al., 2008). Treatment of these tumors with the first-generation ALK inhibitor crizotinib (Xalkori) has shown superior progression-free survival and better overall response compared to treatment with chemotherapy as first- and second-line therapy (Shaw et al., 2013; Solomon et al., 2014). Unfortunately, resistance to crizotinib can develop within a year of treatment and can present with new brain metastases due to the poor penetration of crizotinib across the blood–brain barrier (Costa et al., 2015). Other mechanisms of resistance such as amplification of *ALK*, secondary mutations in *ALK*, and *ALK* independent signaling can play a role in the progression of disease with crizotinib. These shortcomings of therapy have led to the development of new ALK inhibitors: alectinib (Alecensa), ceritinib (Zykadia), and brigatinib (Alunbrig). This article will focus on the newest ALK inhibitors, alectinib and brigatinib.

## PHARMACOLOGY AND MECHANISM OF ACTION

Alectinib is an orally bioavailable tyrosine kinase inhibitor that inhibits ALK and RET proteins by preventing their phosphorylation. Inhibition of ALK activation prevents downstream signaling of cell proliferation and decreases tumor survivability. Alectinib has fivefold more potency inhibiting ALK than crizotinib and maintains activity against many of the secondary mutants associated with resistance to crizotinib. Alectinib has very good blood-brain barrier penetration, with measured concentrations in the blood–cerebrospinal fluid (CSF) approximately equal to the free concentration of alectinib in plasma. It is metabolized via hepatic CYP3A4 enzymes to M4, an active metabolite also metabolized by CYP3A4. It is excreted primarily via the feces (98%) with minimal renal excretion. The mean terminal half-life of alectinib and its active metabolite is about 30 hours (Genentech, 2016).

Brigatinib is another tyrosine kinase inhibitor tested in phase I and II trials which has potent, selective activity inhibiting ALK and ROS1. Brigatinib has 12-fold more potency inhibiting ALK than crizotinib, and it inhibits 17 crizotinib-, ceritinib-, and alectinib-resistant ALK mutants. Brigatinib also has activity against *EGFR* mutations, including the T790M mutation, which is the mutation most often responsible for resistance to first-generation EGFR inhibitors. Taken together, this increased potency and activity against secondary *ALK* mutations make brigatinib a promising treatment in development for *ALK*-mutated NSCLC (Zhang et al., 2016).

## CLINICAL TRIALS

A multicenter, single-arm, phase I/II study conducted in Japan showed the benefit of alectinib in *ALK* rearrangement–positive NSCLC in patients who had not had previous ALK inhibitor exposure (Seto et al., 2013). Of the 46 subjects who received treatment, 44 (93.5%) had an objective response, with 2 subjects (4.3%) having a complete response and 41 subjects (89.1%) with a partial response. Additionally, of the 15 subjects with brain metastasis, none had any reported progression of central nervous system (CNS) lesions at the time of data cutoff, suggesting that alectinib had a benefit in the treatment of CNS disease.

A second phase I study conducted in the United States showed the benefit of alectinib in subjects with crizotinib-resistant NSCLC (Gadgeel et al., 2014). Objective responses were noted in 24 of the 44 subjects (55%) and in 11 of 21 patients with CNS metastases.

Two additional single-arm phase II trials confirmed the activity of alectinib in crizotinib-resistant NSCLC. Shaw and colleagues showed an objective response in 40 of 87 subjects (46%) with an estimated median progression-free survival of 8.1 months (95% confidence interval [CI] = 6.2–12.6 months; Shaw et al., 2016). Of the 16 subjects with measurable CNS disease at baseline, 75% achieved intracranial objective response with a median duration of CNS response of 11.1 months (95% CI = 5.8–11.1 months). Ou and colleagues showed a similar objective response rate (45%) and progression-free survival (8.9 months) as well as CNS disease control rate of 83% and median CNS duration of response of 10.3 months (Ou et al., 2016).

Alectinib efficacy as first-line treatment in NCSLC was recently assessed in 303 subjects in the ALEX trial (Peters et al., 2017). In this open-label, randomized phase III trial, alectinib was compared with crizotinib in patients with ALK rearrangement–positive advanced NSCLC who were naive to treatment. After a median follow-up duration of 17.6 months in the crizotinib arm and 18.6 months in the alectinib arm, the median progression-free survival statistically and clinically favored alectinib (not reached vs. 11.1 months, *p* < .001). Overall CNS progression at 12 months was also found to be significantly lower in the alectinib arm (9.4% vs. 41.4%). Finally, subjects receiving alectinib reported lower rates of adverse events commonly associated with crizotinib treatment, including nausea (14% vs. 48%), vomiting (7% vs. 38%), and diarrhea (12% vs. 45%).

Recently, a phase I/II trial with brigatinib in *ALK*-rearranged NSCLC and other malignancies was performed in 137 subjects (Gettinger et al., 2016). Dose-limiting toxicities in the phase I dose escalation included grade 3 increases in liver enzymes (240 mg daily) and grade 4 dyspnea (300 mg daily). Brigatinib at 180 mg daily was originally chosen as the recommended phase II dose. However, other doses were also evaluated (90 mg once daily and 180 mg once daily with a 7-day lead-in at 90 mg) due to early pulmonary toxicity that was documented in patients starting at 180 mg daily. Phase II expansion cohorts included NSCLC subjects who were both crizotinib naive and crizotinib pretreated. Fifty-two of 79 (66%) subjects with *ALK*-rearranged NSCLC had a confirmed objective response, and all eight of eight patients who were crizotinib naive (100%) had a confirmed objective response. Subjects previously treated with crizotinib (n = 71) had a similar objective response rate of 62%. Eight of 15 subjects with assessable brain lesions had an intracranial response (53%), suggesting a possible role for brigatinib in patients with brain metastases. Median treatment duration of all subjects in the study, including those without *ALK*-rearranged NSCLC, was 7.5 months, and the median duration of treatment was 15.4 months among those in the study with *ALK*-rearranged NSCLC.

Subsequent to the phase I/II trial published by Gettinger and colleagues, a randomized phase II trial of brigatinib in crizotinib-refractory, *ALK*-positive NSCLC enrolled 112 subjects to receive brigatinib at 90 mg once daily (arm A) and 110 subjects to receive brigatinib at 180 mg daily with a 7-day lead-in at 90 mg daily (arm B; Kim et al., 2017). Investigator-assessed objective response rates were 45% and 54% in arm A and arm B respectively, and progression-free survival was 9.2 months in arm A and 12.9 months in arm B, a statistically significant finding favoring dosing with 180 mg daily with a 7-day lead-in at 90 mg daily. Intracranial efficacy was also demonstrated with an objective intracranial response in 42% of subjects in arm A and 67% in arm B. Preliminary overall survival estimates showed 1-year overall survival in 71% of subjects in arm A and 80% of subjects in arm B.

## ADVERSE EFFECTS

In the two phase II trials with alectinib, the most common adverse events were constipation (11%–32%), fatigue (25%–30%), myalgia (17%–22%), and peripheral edema (15%–24%; Ou et al., 2016; Shaw et al., 2016). Other adverse events included aspartate aminotransferase (AST)/alanine aminotransferase (ALT) elevation, creatine phosphokinase (CPK) elevation, nausea, diarrhea, and rash. Grade 3 and 4 adverse events were uncommon, with the most common being reduced neutrophil count (4%), dyspnea (2%), and elevations in liver enzymes (2%) or CPK (4%).

In the phase I/II trial with brigatinib, the most common treatment-related adverse effects included nausea (53%), fatigue (43%), and diarrhea (41%), mostly of grade 1 or grade 2 in severity (Gettinger et al., 2016). The most common grade 3 and grade 4 treatment-related adverse effects included increased lipase concentration (9%), dyspnea (6%), and hypertension (5%). Serious treatment-related adverse effects occurring in > 5% of subjects, included dyspnea (7%), pneumonia (7%), and hypoxia (5%). Early-onset pulmonary adverse events led to the investigation of different dosages within the phase II expansion cohorts (90 mg once daily and 180 mg once daily with a 7-day lead-in at 90 mg). Interestingly, none of the 32 patients treated with 180 mg once daily with a 7-day lead-in had an early-onset pulmonary event after escalation to 180 mg once daily. Dose reductions were recorded in 15% of patients who received recommended phase II doses.

In the randomized phase II trial with brigatinib, the most common any-grade treatment-emergent adverse events were nausea (33% vs. 40%), headache (28% vs. 27%), and diarrhea (19% vs. 38%) in arm A vs. arm B, respectively (Kim et al., 2017). The most common grade ≥ 3 treatment-emergent adverse events were hypertension (6% vs. 6%), increased CPK (3% vs. 9%), pneumonia (3% vs. 5%), and increased lipase (4% vs. 3%) in arm A vs. arm B, respectively. Pulmonary adverse events with early onset were seen in 6% of all patients in the randomized phase II study, and these early-onset pulmonary adverse events were only seen at doses of 90 mg in both arms. No early-onset pulmonary adverse events were seen after escalation to 180 mg daily. These early-onset pulmonary adverse events were managed with dose interruption and subsequent reintroduction at 60 mg daily in 6 of 14 patients.

ALK inhibitors as a class are associated with bradycardia and a more serious but rare side effect, interstitial lung disease. See Table 1 and Table 2 for how to manage dose modifications related to adverse events.

**Table 1:**
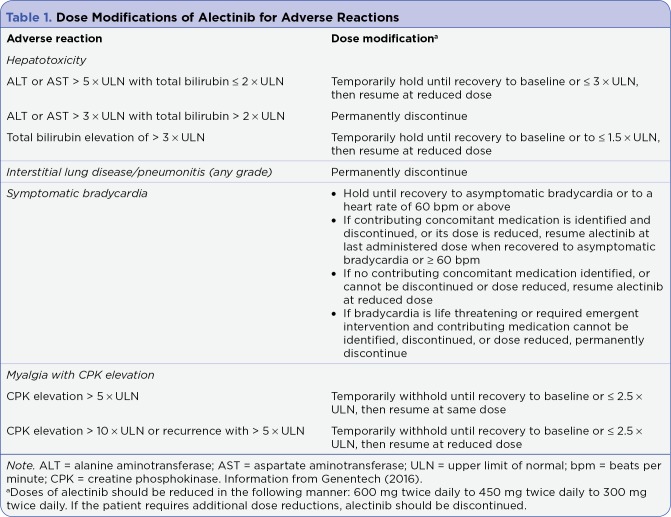
Dose Modifications of Alectinib for Adverse Reactions

**Table 2:**
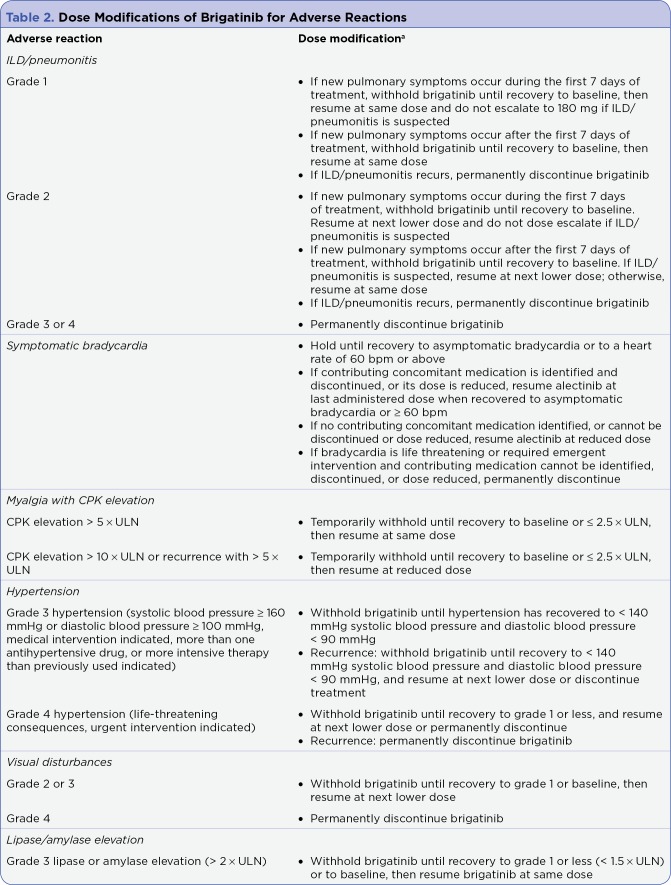
Dose Modifications of Brigatinib for Adverse Reactions

**Table 2:**
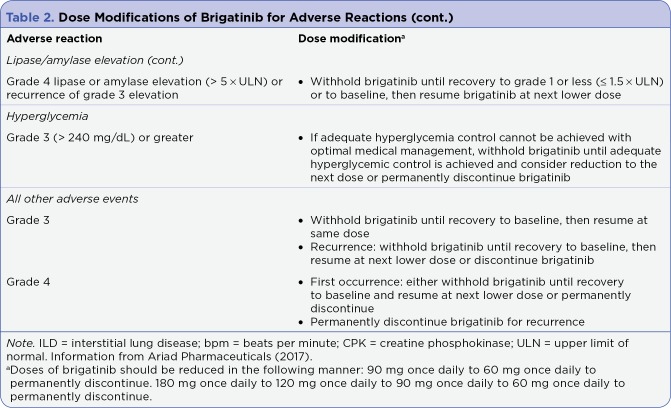
Dose Modifications of Brigatinib for Adverse Reactions (cont.)

## ROLE IN THERAPY

With recent US Food and Drug Administration (FDA) approvals for ALK-, EGFR-, and ROS1-targeted medications and PD-1/PD-L1–directed immunotherapy, the options available for the treatment of advanced or metastatic NSCLC have expanded. Therapy options such as ALK inhibitors, which target specific mutations associated with a patient’s cancer, provide an alternative option to cytotoxic chemotherapy.

Crizotinib has been approved for use in *ALK*-positive metastatic NSCLC since 2013. Whereas crizotinib therapy provides durable responses lasting about a year, ceritinib, alectinib, and brigatinib are indicated as second-line treatment after a patient’s *ALK*-positive NSCLC has developed resistance to crizotinib. Additionally, alectinib and brigatinib have data showing efficacy in patients with documented brain metastases.

As shown in phase II trials, alectinib, with an objective response rate of 45% and median progression-free survival of 8 to 12 months, provides an additional treatment option after a patient has developed CNS metastases and/or resistance to crizotinib. Additionally, the ALEX trial shows that alectinib is an effective option in the treatment-naive setting for *ALK*-rearranged NSCLC. Finally, based on clinical trial data, alectinib is a well-tolerated medication with low rates of grade 3 or 4 adverse events.

Brigatinib has also been shown to be an effective and safe option in the second-line setting for patients with *ALK*-positive NSCLC after progression on crizotinib. Ongoing clinical trials will evaluate the use of brigatinib for treatment-naive *ALK*-positive NSCLC patients, and there is also a trial evaluating the use of brigatinib after disease progression on second-generation ALK inhibitors (ceritinib and alectinib), which is currently accruing patients.

## IMPLICATIONS FOR THE ADVANCED PRACTITIONER

Although the incidence of *ALK*-positive NCSLC only accounts for 5% of all NCSLC cases, the efficacy of tyrosine kinase inhibitors with ALK activity ensures that these agents will have a place in the treatment of advanced and metastatic disease. Given that patients with adenocarcinoma and large cell histologies with NSCLC more commonly have genetic driver mutations than squamous cell carcinoma, patients with newly diagnosed nonsquamous NSCLC should routinely undergo genetic testing of their tumors for driver mutations in *ALK*, *EGFR*, and *ROS1*. Genetic testing of squamous cell NSCLC is not routinely performed unless patients have characteristics associated with genetic aberrations in *ALK*, *EGFR*, or *ROS1* such as young age or nonsmoking patients. As patients with *ALK*-positive NSCLC are identified and started on treatment, it is important that the advanced practitioner in oncology be aware of the benefits and risks associated with the newest ALK-active tyrosine kinase inhibitors.

The recommended dose of alectinib is 600 mg twice daily. Administration with a high-fat meal is associated with a 3.1-fold increase in absorption. Therefore, patients should be instructed to take alectinib with food. No dose reductions are recommended for baseline renal and hepatic dysfunction, although alectinib has not been studied in patients with creatinine clearance < 30 or moderate to severe hepatic impairment (Genentech, 2016).

Adverse events associated with alectinib are usually mild and discontinuation of therapy occurred in < 10% of patients in phase II clinical trials. Constipation, fatigue, and myalgia were the most common adverse events, but AST/ALT elevations, hyperbilirubinemia, and CPK elevations have been observed in the first few months of therapy. As a result, it is recommended by the prescribing information to monitor AST/ALT and serum bilirubin every 2 weeks for the first 3 months and CPK every 2 weeks for the first month of therapy. Table 1 has recommendations for dose modifications related to adverse events of alectinib.

Although alectinib is a substrate of CYP3A4, no clinically meaningful interactions were observed when it was administered with strong 3A4 inhibitors (posaconazole) or inducers (rifampin).

Treatment with alectinib is associated with a high cost of therapy (average wholesale price is $14,792.90 for a 30-day supply) and patient assistance programs should be considered for all patients without financial means to support long-term therapy. Brigatinib is also a high-cost therapy with an average wholesale price of $17,955 for a 30-day supply. There are also patient assistance programs available for brigatinib, which should be utilized for patients struggling to afford their copays.

Brigatinib should be prescribed at a dosage of 90 mg daily for the first 7 days with dose-escalation to 180 mg daily thereafter if tolerated. There are no recommended dose adjustments for hepatic or renal impairment, but brigatinib has not been studied in patients with moderate to severe hepatic impairment or severe renal impairment (Ariad Pharmaceuticals, 2017).

Adverse events with brigatinib in clinical trials have generally been reported as mild, with nausea, diarrhea, fatigue, cough, headache, rash, and hypertension being reported as grade 1 or grade 2 in 20% to 30% of patients. Additionally, brigatinib carries a warning of hyperglycemia, pancreatic enzyme elevation, and CPK elevation. Patients should be monitored at baseline and periodically for elevations in CPK, pancreatic enzymes, and liver enzymes. Bradycardia is a class effect of ALK inhibitors, and patients with symptomatic bradycardia should be evaluated for other offending medications (beta blockers), and dose adjustment may be required. Early-onset pulmonary adverse events and pneumonitis are rarer adverse events that should be monitored for closely during the first several weeks of treatment. Dose adjustment or dose interruption may be necessary for patients experiencing early-onset pulmonary adverse events. See Table 2 for dose adjustment criteria for brigatinib.

Brigatinib is a substrate and inducer of CYP3A4 enzymes, and it should not be coadministered with strong CYP3A4 inducers, inhibitors, or grapefruit products. Since brigatinib is a CYP3A4 inducer, it may decrease concentrations of CYP3A4 substrates such as oral contraceptives, rendering them ineffective.

## CONCLUSION

Alectinib and brigatinib are the newest second-generation ALK inhibitors that show efficacy in patients with *ALK*-positive NSCLC whose disease has progressed on the first-generation ALK inhibitor crizotinib. The ALEX trial showed superior efficacy and safety results with alectinib compared to crizotinib in the treatment-naive setting for *ALK*-rearranged NSCLC. Although it appears that a durable response is achievable with both of these agents, patients with NSCLC treated with these agents will eventually have disease progression. Further innovation with third-generation ALK inhibitors and next-generation tumor sequencing will allow practitioners to further tailor treatment to patients’ individual tumor genetics. These treatments promise hope for improvement in overall survival for *ALK*-positive NSCLC patients.
